# Relative ellipsoid zone reflectivity and its association with disease severity in age-related macular degeneration: a MACUSTAR study report

**DOI:** 10.1038/s41598-022-18875-5

**Published:** 2022-09-02

**Authors:** Marlene Saßmannshausen, Charlotte Behning, Ben Isselmann, Matthias Schmid, Robert P. Finger, Frank G. Holz, Steffen Schmitz-Valckenberg, Maximilian Pfau, MACUSTAR Consortium, H. Agostini, L. Altay, R. Atia, F. Bandello, P. G. Basile, C. Behning, M. Belmouhand, M. Berger, A. Binns, C. J. F. Boon, M. Böttger, C. Bouchet, J. E. Brazier, T. Butt, C. Carapezzi, J. Carlton, A. Carneiro, A. Charil, R. Coimbra, M. Cozzi, D. P. Crabb, J. Cunha-Vaz, C. Dahlke, L. de Sisternes, H. Dunbar, R. P. Finger, E. Fletcher, H. Floyd, C. Francisco, M. Gutfleisch, R. Hogg, F. G. Holz, C. B. Hoyng, A. Kilani, J. Krätzschmar, L. Kühlewein, M. Larsen, S. Leal, Y. T. E. Lechanteur, U. F. O. Luhmann, A. Lüning, I. Marques, C. Martinho, G. Montesano, Z. Mulyukov, M. Paques, B. Parodi, M. Parravano, S. Penas, T. Peters, T. Peto, M. Pfau, S. Poor, S. Priglinger, D. Rowen, G. S. Rubin, J. Sahel, C. Sánchez, O. Sander, M. Saßmannshausen, M. Schmid, S. Schmitz-Valckenberg, H. Schrinner-Fenske, J. Siedlecki, R. Silva, A. Skelly, E. Souied, G. Staurenghi, L. Stöhr, D. J. Taylor, J. H. Terheyden, S. Thiele, A. Tufail, M. Varano, L. Vieweg, L. Wintergerst, A. Wolf, N. Zakaria, Sarah Thiele

**Affiliations:** 1 Department of Ophthalmology, University of Bonn, Venusberg-Campus 1, 53127 Bonn, Germany; 2 GRADE Reading Center, University of Bonn, Bonn, Germany; 3 Institute for Medical Biometry, Informatics and Epidemiology, University of Bonn, Bonn, Germany; 4 John A. Moran Eye Center, Department of Ophthalmology and Visual Sciences, University of Utah, Salt Lake City, UT USA; 5 Ophthalmic Genetics and Visual Function Branch, National Eye Institute, Bethesda, MD USA; 6 Universitaetsklinikum Freiburg (UKLFR), Department of Ophthalmology, University of Freiburg, Freiburg, Germany; 7 Department of Ophthalmology, University Hospital of Cologne, Cologne, Germany; 8 Quinze-Vingts National Ophthalmology Hospital, UPMC-Sorbonne Université, Paris, France; 9 Department of Ophthalmology, University Vita Salute-Scientific Institute of San Raffael, Milan, Italy; 10 AIBILI Association for Innovation and Biomedical Research on Light and Image (AIBILI), Coimbra, Portugal; 11 Department of Ophthalmology, Rigshospitalet-Glostrup, Copenhagen University, Glostrup, Denmark; 12 City University London, London, UK; 13 Department of Ophthalmology, Leiden University Medical Center, Leiden, The Netherlands; 14 BAYER AG, Leverkusen, Germany; 15 Novartis Pharma AG, Basel, Switzerland; 16 University of Sheffield, Sheffield, UK; 17 University College London (UCL), London, UK; 18 Fondation Voir et Etendre, Paris, France; 19 Centro Hospitalar de Sao Joao EPE (Hospital Sao Joao), Department of Ophthalmology, Porto Medical School, Porto, Portugal; 20 Department of Ophthalmology Luigi Sacco Hospital, University of Milan, Milan, Italy; 21 Carl Zeiss Meditec, AG, Jena, Germany; 22 Clinical Trial Unit, Department of Ophthalmology, Gloucestershire Hospitals NHS Foundation Trust, Cheltenham, UK; 23 Department of Ophthalmology, St. Franziskus Hospital, Münster, Germany; 24 Ophthalmology and Vision Sciences, The Queen’s University and Royal Group of Hospitals Trust, Belfast, Northern Ireland, UK; 25 Stichting Katholieke Universiteit/Radboud University Medical Center (SRUMC), Radbound University, Nijmegen Medical Center, Nijmegen, The Netherlands; 26 Department of Ophthalmology, University of Ulm, Ulm, Germany; 27 STZ Biomed and STZ Eyetrial at the Center of Ophthalmology, University Hospital Tuebingen, Tübingen, Germany; 28 F. Hoffmann-La Roche Ltd, Basel, Switzerland; 29 Centre Hospitalier National d’Opthalmologie des Quinze-Vingts, Paris, France; 30 G. B. Bietti Eye Foundation-IRCCS, Rome, Italy; 31 Ludwig-Maximilians-Universitaet Muenchen (LMU), University Eye Hospital, Munich, Germany; 32 European Clinical Research Infrastructure Network (ECRIN), Paris, France; 33 Centre Hospitalier Intercommunal de Creteil (HIC), University Eye Clinic, Centre Hospitalier Creteil, Paris, France; 34 Moorfields Eye Hospital NHS Foundation Trust (MBRC), London, UK

**Keywords:** Eye diseases, Diseases

## Abstract

Quantification of the relative ellipsoid zone reflectivity (rEZR) might be a structural surrogate parameter for an early disease progression in the context of age-related macular degeneration (AMD). Within the European multicenter, cross-sectional MACUSTAR study, we have devised an automatic approach to determine the mean rEZR [arbitrary units, AU] at two independent visits in SD-OCT volume scans in study participants. Linear mixed-effects models were applied to analyze the association of AMD stage and AMD associated high-risk features including presence of pigmentary abnormalities, reticular pseudodrusen (RPD), volume of the retinal-pigment-epithelial–drusenoid-complex (RPEDC) with the rEZR. Intra-class correlation coefficients (ICC) were determined for rEZR reliability analysis. Within the overall study cohort (301 participants), we could observe decreased rEZR values (coefficient estimate ± standard error) of − 8.05 ± 2.44 AU (p = 0.0011) in the intermediate and of − 22.35 ± 3.28 AU (p < 0.0001) in the late AMD group. RPD presence was significantly associated with the rEZR in iAMD eyes (− 6.49 ± 3.14 AU; p = 0.0403), while there was a good ICC of 0.846 (95% confidence interval: 0.809; 0.876) in the overall study cohort. This study showed an association of rEZR with increasing disease severity and the presence of iAMD high-risk features. Further studies are necessary to evaluate the rEZR’s value as a novel biomarker for AMD and disease progression.

## Introduction

With emerging therapies for early and intermediate age-related macular degeneration (iAMD) on the horizon, the identification of valid biomarkers which reliably detect and or predict disease progression and might serve as structural outcome measures in interventional clinical trials is essential^1–4^. Although iAMD features, i.e. large drusen and pigmentary abnormalities (PA), are known to confer risk for development of advanced disease stages, their assessment alone may not be sensitive enough for reliable determination of progression probabilities in earlier stages of the disease^5–7^.

Growing evidence suggests that mitochondrial dysfunction plays a key role in the pathophysiology of outer retinal degeneration in the context of AMD^2^. Current understanding states that photoreceptor mitochondria are responsible for the ellipsoid zone (EZ) signal in spectral-domain optical coherence tomography (SD-OCT)^8–12^. Based on this the quantification of the relative ellipsoid zone reflectivity (rEZR) may be a structural surrogate of the axial compartmentalization and transverse alignment of photoreceptors, and thus an indicator of outer retinal integrity and health ("functional imaging")^8–10,13^. In fact, previous studies found the rEZR to be significantly reduced in subjects with iAMD and associated with the presence of established high-risk features for iAMD progression and choriocapillaris flow impairment^14–16^. Against the background of AMD as a chronic-progressive disease with increasing degenerative alterations and loss of photoreceptors over time, a rEZR decrease across different AMD stages would further support its relevance as a candidate biomarker for mitochondrial dysfunction in the outer retina^17–19^. However, a systematic analysis of the rEZR in different AMD stages has not been performed yet.

For acceptance by regulatory agencies, novel biomarkers are required to demonstrate pathophysiological relevance and be both feasible and reliable. With now available automated approaches, rEZR determination is feasible in large data sets but its reliability remains to be assessed^15,20–22^.

The purpose of this work is to refine the characterization of rEZR as, apart from established and AMD-typical drusen, a novel biomarker for photoreceptor dysfunction in AMD subjects. Here, the impact of AMD staging and established iAMD high-risk structural features on the rEZR as well as the rEZR inter-session reliability will be analyzed across different AMD stages. This study is performed within the prospectively acquired data set of the MACUSTAR trial, which is a multicenter European, low-interventional clinical study aiming to identify and validate new clinical endpoints, like rEZR, for future clinical interventional trials in iAMD^20–22^.

## Results

### Baseline characteristics

A total of 301 eyes of 301 subjects (female n = 187; 62.1%) with a mean age of 71.2 ± 7.2 years were enrolled. Out of those, 34 (11.3%) patients were categorized as early, 168 (55.8%) as intermediate and 43 (14.3%) patients as late-stage AMD, while 56 (18.6%) of the included subjects were controls. The mean age of the study patients in the early AMD subgroup was 71.7 ± 6.38 years, in the iAMD subgroup 71.2 ± 7.55 years, and in the late AMD group 74.9 ± 5.59 years.

Due to insufficient image quality of SD-OCT image data at V1, one iAMD study subject was excluded from the analyses leading to a total of 167 assessed subjects in the iAMD subgroup.

At V1, the mean rEZR in the overall AMD study population was 34.3 ± 17.9 AU with a mean rEZR of 42.0 ± 18.9 AU in the early AMD group, 37.0 ± 16.8 AU in participants with iAMD and 17.7 ± 9.5 AU with late AMD, respectively. In contrast, healthy subjects exhibited a higher rEZR with 47.6 ± 20.2 AU. Please see Table 1 for further descriptive characteristics of the overall rEZR assessed study cohort.

**Table 1:** Characteristics at baseline for the study population eligible for rEZR determination (controls and AMD subjects), for only controls, for all AMD subjects and for each of the AMD subgroups.

	Overall Study population (n = 300)	Controls (n = 56)	All AMD (n = 244)	Early AMD (n = 34)	iAMD (n = 167)	Late AMD (n = 43)
**Age [years]**						
Mean ± SD	71.2 ± 7.21	68.1 ± 6.35	71.9 ± 7.21	71.7 ± 6.38	71.2 ± 7.57	74.9 ± 5.59
Median [min, max]	72.0 [55.0, 88.0]	68.9 [55.0, 80.0]	72.0 [55.0, 88.0]	72.0 [57.0, 82.0]	72.0 [55.0, 88.0]	68.0 [55.0, 80.0]
**Gender [female]**						
n (%)	186 (62.0%)	33 (58.9%)	153 (62.7%)	27 (79.4%)	105 (62.9%)	21 (48.8%)
**BCVA [logMAR]**						
Mean ± SD	0.12 ± 0.30	− 0.04 ± 0.08	0.15 ± 0.32	0.01 ± 0.08	0.02 ± 0.10	0.77 ± 0.25
Median [min, max]	0.02 [− 0.24, 1.24]	− 0.06 [− 0.24, 0.14]	0.04 [− 0.24, 1.24]	0.02 [− 0.18, 0.20]	0.02 [− 0.24, 0.68]	0.84 [0.20, 1.24]
**rEZR [AU]**						
Mean ± SD	36.8 ± 19.0	47.6 ± 20.2	34.3 ± 17.9	42.0 ± 18.9	37.0 ± 16.8	17.7 ± 9.54
Median [min, max]	34.3 [8.03, 108]	43.1 [11.0, 108]	14.1 [8.03, 99.2]	34.8 [16.7, 99.2]	34.6 [8.94, 91.0]	14.1 [8.03, 50.7]

*AMD* age-related macular degeneration, *iAMD* intermediate AMD, *AU* arbitrary units, *BVCA* best-corrected visual acuity, *SD* standard deviation, *rEZR* relative ellipsoid zone reflectivity.

### Impact of AMD severity stage on the rEZR

**Figure 1:**
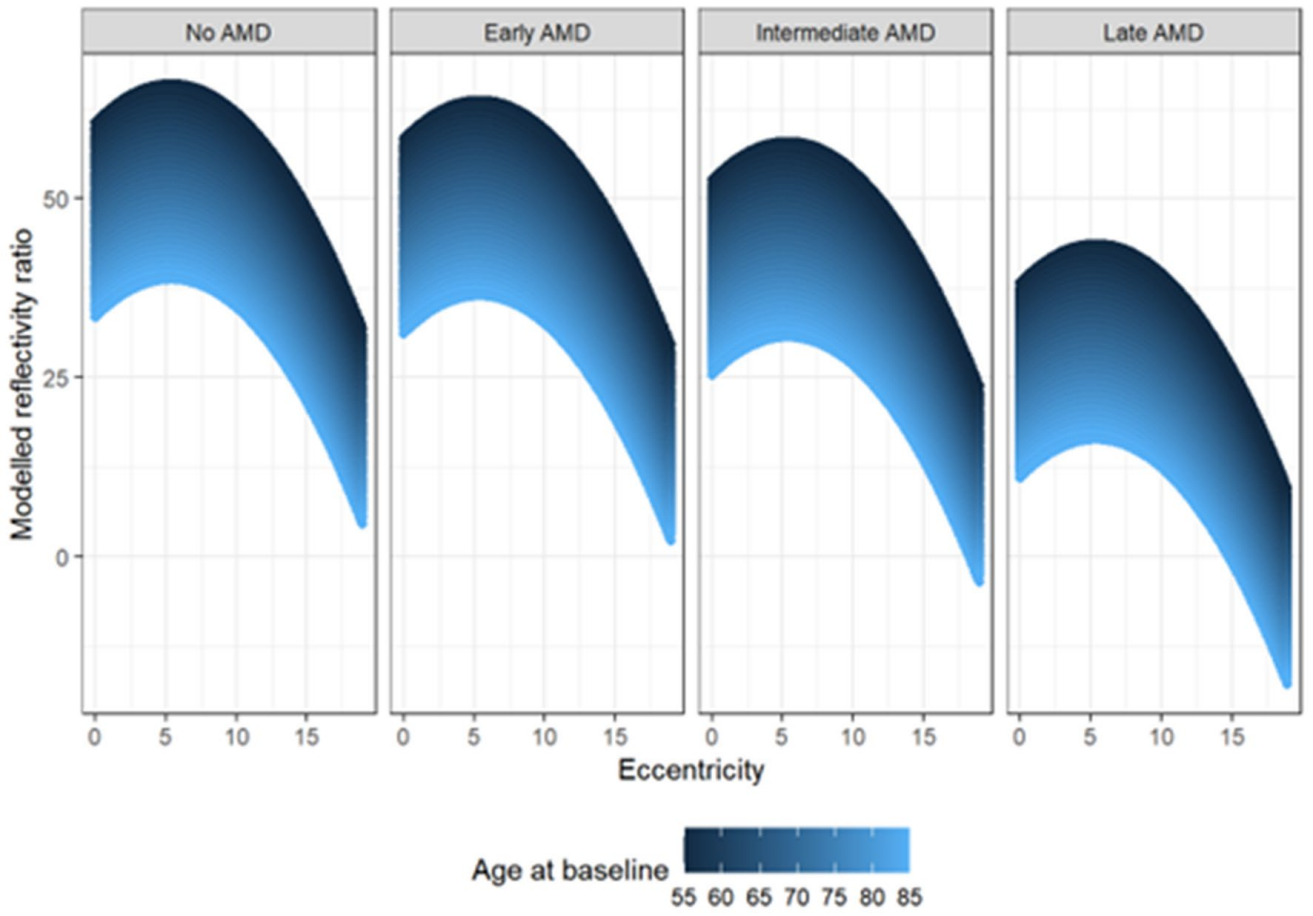
Graphical representation of linear mixed-effects model analysis with the rEZR as dependent variable and the age at baseline (color-coded in blue: younger age to older age = dark blue to light blue), as well as the topographic variation (eccentricity [°]) of the rEZR from the fovea within each SD-OCT raster scan differentiated for each study group (controls, early AMD, intermediate AMD and late-stage AMD). Note, linear mixed-effects model showed a decreased rEZR in AMD subjects compared to healthy individuals with overall lowest rEZR in the late AMD group followed by the intermediate AMD group. Further, the rEZR was found to first increase and then decrease with increasing eccentricity respecting highest rEZR values at approximately 5° perifoveally within each subgroup. In addition, the rEZR showed an association with subjects’ age at baseline indicated by lower modelled rEZR values in older AMD subjects and controls. *AMD* age-related macular degeneration.

**Figure 2:**
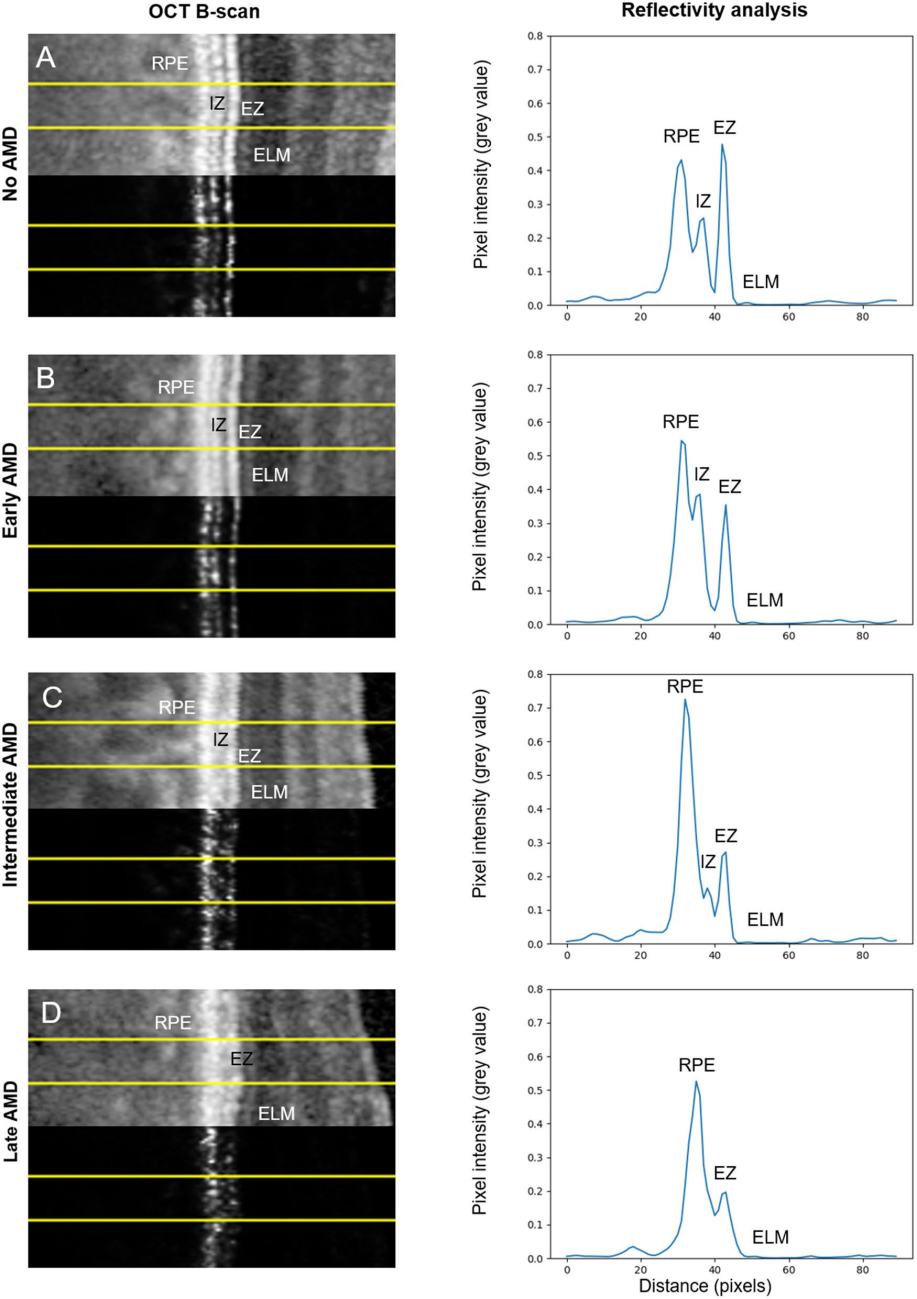
SD-OCT image details (pixel intensities displayed: logarithmic transformed (above) and raw (below)) of representative cases for (**A**) healthy controls, (**B**) early AMD, (**C**) intermediate AMD and (**D**) late AMD and their corresponding reflectivity profiles. The yellow superimposed yellow lines indicate the region of interest (ROI) on the logarithmic (above) and the linear (below) displayed OCT scan. Note, while the logarithmic displayed OCT image is presented for better visualization of outer retinal structure, all actual rEZR analyses in this study were performed in the image raw data (linear displayed).

In a linear mixed-model correcting for age, gender, and topographic dependence of the rEZR within the SD-OCT raster scan, patients with iAMD and late AMD exhibited a significant decreased rEZR with a coefficient estimate (± SE) of − 8.05 ± 2.44 AU (p = 0.0011) and − 22.35 ± 3.28 AU (p < 0.0001), respectively, compared to healthy controls. Further, subject age had a significant impact on rEZR with a decrease of − 0.87 ± 0.13 AU (p < 0.0001) per year. The topographic variation was included as a spline function of the distance from the foveal center, and its association rEZR is depicted in Fig. 1. Table 2 summarizes the linear-mixed model results of AMD staging and its impact on rEZR. Figure 2 demonstrates reflectivity profiles of representative cases for each study subgroup.

**Table 2:** Results of multivariable linear mixed-model analysis of the impact of AMD severity stage compared to control on the rEZR as presented with coefficient estimates, standard error and p-value, respectively. Eccentricity is included in the model as a b-spline of degree 2.

	Coefficient estimates	Standard error	p-value
(Intercept)	107.2	9.08	
Early AMD	− 2.29	3.45	0.5078
Intermediate AMD	− 8.05	2.44	0.0011
Late AMD	− 22.35	3.28	< 0.001
Age at baseline [years]	− 0.87	0.13	< 0.001
Gender [male]	1.17	1.90	0.538
bs (Eccentricity) [°]	− 28.78	0.085	< 0.001

*AMD* age-related macular degeneration.

### Impact of iAMD high-risk features on the rEZR

Out of the 167 eyes of 167 iAMD subjects, RPD were present in 22.2% (n = 37), PED in 4.8% (n = 8), PA in 56.9% (n = 95), vitelliform material in 4.2% (n = 7), GA in the fellow eye in 3.0% (n = 5), refractile deposits in 3.0% (n = 5), qCNV in 2.4% (n = 4) and mean RPEDC volume was 0.0392 ± 0.0690 mm^3^. Separate linear mixed models were applied to consider the individual association of structural parameters on rEZR in the study eye of iAMD subjects. In detail, the presence of RPD and PA was associated with a lower rEZR with coefficient estimates of − 8.84 ± 2.92 (p = 0.0028) and − 6.08 ± 2.34 (p = 0.0104) respectively. In contrast, no association was found for the presence of PED, vitelliform material, GA in the fellow eye or with RPEDC volume (Table 3). For a detailed presentation of the linear-mixed model analyses with one structural parameter each, please see Supplemental Table S1.

**Table 3:** Results of separate linear mixed-model analyses of the impact of iAMD high-risk features on the rEZR in eyes with iAMD as presented with coefficient estimates, standard error and p-value, respectively.

	Coefficient estimates	Standard error	p-value
Presence of RPD*	− 8.84	2.92	0.0028
Presence of PED*	− 9.63	5.57	0.0855
Presence of GA in FE*	− 10.49	6.34	0.0998
Presence of PA*	− 6.08	2.34	0.0104
Presence of vitelliform material*	− 7.89	5.93	0.186
Presence of refractile deposits*	− 10.51	6.93	0.131
Presence of qCNV*	− 13.27	7.74	0.0885
RPEDC volume [mm^3^]*	− 24.52	17.35	0.159

*Each separate linear mixed-model was adjusted for patient’s age, gender and the eccentricity.

*iAMD* intermediate age-related macular degeneration, *RPD* reticular pseudodrusen, *PED* pigment-epithelium detachment, *GA* geographic atrophy, *FE* fellow eye, *PA* pigmentary abnormalities, *qCNV* quiescent choroidal neovascularization, *RPEDC* retinal-pigment-epithelium complex volume.

In multivariable analysis assessing the impact of all structural parameters on the rEZR, presence of RPD was revealed to be the only structural feature exhibiting an association with rEZR with a coefficient estimate of − 6.49 ± 3.14 (p = 0.0403). Results of the multivariate analysis are shown in Table 4.

**Table 4:** Results of a combined multivariable linear mixed-model analysis of the impact of iAMD high-risk features on the rEZR in eyes with iAMD as presented with coefficient estimates, standard error and p-value, respectively.

	Coefficient estimates	Standard error	p-value
(Intercept)	90.62	11.73	
Age at baseline [years]	− 0.80	0.17	< 0.001
Gender [male]	3.44	2.54	0.1769
Presence of RPD	− 6.49	3.14	0.0403
Presence of PED	− 9.00	6.80	0.1876
Presence of GA in FE	− 7.23	6.39	0.2599
Presence of PA	− 2.70	2.45	0.2731
Presence of vitelliform lesion	− 3.78	6.27	0.5473
Presence of refractile deposits	− 8.23	6.83	0.2304
Presence of qCNV	− 5.61	8.88	0.5290
RPEDC volume [mm^3^]	− 8.89	27.11	0.7433
bs(Eccentricity) [°]			< 0.001

*iAMD* intermediate age-related macular degeneration, *RPD* reticular pseudodrusen, *PED* pigment-epithelium detachment, *GA* geographic atrophy, *FE* fellow eye, *PA* pigmentary abnormalities, *qCNV* quiescent choroidal neovascularization, *RPEDC* retinal-pigment-epithelium complex. Eccentricity is included as a b-spline with degree 2. The full models can be found in the supplementary material.

### Inter-session reliability of the rEZR

Eleven study eyes (no AMD n = 2; early AMD n = 6; iAMD n = 1; late AMD n = 2) were excluded from this analysis as no data were available for follow-up. For another 4 eyes (no AMD n = 1, iAMD n = 3), the rEZR could only be determined at one visit due to missing data or image data with insufficient quality. For the remaining 286 study eyes, mean time of retinal imaging data acquisition between V1 and V3 was 37.7 ± 18.1 (range, 15–189) days.

Analysis of the inter-session reliability for the average rEZR showed an interclass correlation coefficient (ICC) of 0.846 (95% CI: 0.809; 0.876) for the overall study population. Subgroup analysis exhibited an ICC of 0.683 (95% CI: 0.426; 0.839) in early, of 0.834 (95% CI: 0.780; 0.875) in intermediate and of 0.936 (95% CI: 0.884; 0.965) in the late AMD subgroup. An ICC of 0.752 (95% CI: 0.607; 0.849) was determined in the control group.

**Figure 3:**
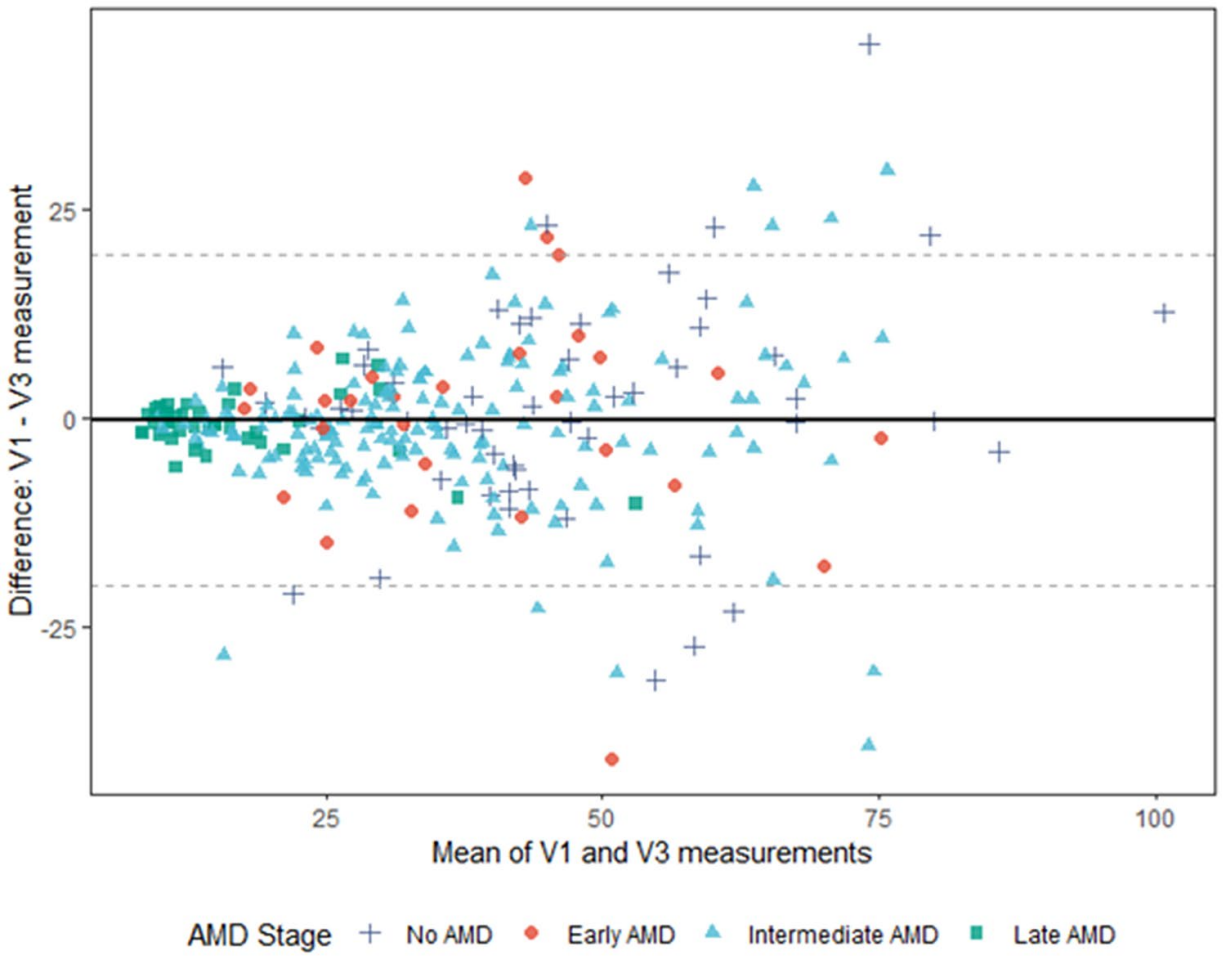
Bland–Altman plot for the overall test–retest study population (n = 286) of the differences in the mean rEZR values per patient at V1 and V3 (solid line: mean difference; dotted lines: mean ± 1.96 × standard deviation). The rEZR difference between both visits (y axis) is presented against the rEZR average for V1 and V3 (x axis). *AMD* age-related macular degeneration, *V1* screening visit, *V3* follow-up visit, *rEZR* relative ellipsoid zone reflectivity.

In addition, there was no evidence for a proportional bias, i.e. a Deming regression between the V1 and V3 measurements yielded a slope estimate (± SD) of − 2.73 ± 1.56 [95% CI: − 5.48; 0.4] and intercept estimate of 1.07 ± 0.06 [95% CI: 0.96; 1.17]. Figure 3 shows a Bland–Altman plot for graphical representation of the test–retest analysis for the overall study population. The Bland–Altman 95% limits of agreement were − 20.02 and 19.60, with an offset close to zero (mean difference ± SD of − 0.21 ± 10.10).

Bland–Altman analysis of the overall study cohort revealed a total of 22 (7.6%) patients with rEZR values outside of the limits of agreement, i.e. above/below the mean difference ± 1.96 × standard deviation between V1 and V3. Out of the 22 subjects, 8 were categorized as healthy individuals, 4 as early AMD and 10 as iAMD. A detailed image review of these cases was performed to identify any potential feature impeding reliable rEZR assessment between V1 and V3. However, neither any AMD-associated structural abnormality nor any technical issue in image acquisition could be identified.

## Discussion

In this study, the rEZR was shown to be associated with AMD staging and iAMD high-risk features demonstrating significant lower rEZR values as disease severity increases. Additionally, reliability of rEZR determination was demonstrated to be good across all disease stages and in healthy controls between two independent study visits. Given the assessment of the EZ and therefore photoreceptor structure was performed in retinal areas without typical sub-RPE drusen, the findings of this study support the current pathophysiological understanding of AMD as a widespread, degenerative retinal disease beyond obviously localized morphological lesions^23^. Moreover, the here presented findings highlight the potential of the rEZR as a novel and innovative structural biomarker for photoreceptor degeneration in AMD.

Results of previous studies also show a significant lower rEZR in AMD subjects compared to age-similar healthy individuals. However, this study is the first to demonstrate that this decrease correlates with AMD stage^14–16^. While early AMD patients showed an overall rEZR decrease of − 2.29 ± 3.45 AU (coefficient estimate ± standard error; p = 0.5078), the decrease was more pronounced in intermediate and late-stage AMD patients (− 8.05 ± 2.44 AU, p = 0.0011 and − 22.35 ± 3.28 AU, p < 0.0001, respectively). These findings are in line with the assumption of wide-spread retinal degenerative alterations in the natural course of AMD, highlighting a potential role of rEZR as a candidate structural biomarker for photoreceptor degeneration in AMD^5,17–19^. Given the unmet need for therapeutic interventions in early AMD stages, rEZR was evaluated in comparison to established iAMD high-risk features for disease progression. Here, iAMD subjects showed associations between the rEZR and the presence of both RPD (p = 0.0028) and PA (p = 0.0104). In multivariable analysis, i.e. when all iAMD high-risk features were considered simultaneously, only the presence of RPD remained significantly associated with the rEZR (p = 0.0403). Interestingly, an increase of RPEDC volume, a quantitative measure for sub-RPE drusen load, showed no significant impact on the rEZR in uni- (p = 0.159) or in multivariable (p = 0.7433) analysis. These findings are in accordance with the results of the first longitudinal rEZR evaluation and its association with iAMD baseline characteristics recently performed by Thiele et al.^24^. While the analysis of Thiele and coworkers was performed in cohort of AMD patients with bilateral large drusen at baseline independent of MACUSTAR, the extent of RPD was also shown to demonstrate a significant impact on both baseline rEZR, as well as on rEZR change over time. Moreover, and again consistent with these MACUSTAR results, RPD was confirmed to be the only iAMD high-risk structural feature remaining statistically significantly associated (p = 0.042) in multivariable analysis. Both drusen volume and PA were not significantly associated (both: p > 0.068). The MACUSTAR findings highlight the growing evidence of RPDs' distinction from sub-RPE drusen within the AMD pathogenesis, also implying a difference in their impact on photoreceptor dysfunction. This has already been demonstrated in several studies assessing retinal function in AMD^25–28^. Based on our results it is conceivable that RPD cause a more pronounced and direct local effect of RPD on photoreceptor metabolism and therefore also on EZ signal in SD-OCT imaging compared to sub-RPE drusen^28–30^. Noteworthy, in this study RPD area was not excluded and therefore it is currently unclear if the reflectivity of the EZ and ELM bands could be (structurally) altered due to physical changes in the band contour directly above RPD lesions or if any reflectivity changes of the EZ and ELM topographically above RPD might be the result of actual pathological changes.

In our study we could demonstrate a good test–retest reliability of rEZR determination across all AMD subgroups and healthy individuals. A further assessment of imaging data from observations outside the Bland–Altman limits of agreement did not identify any structural finding or technical issue in the image itself as a potential problem.

Several limitations need to be considered. This analysis was performed on a cross-sectional data set, and therefore assessment of prognostic value is not possible. Further longitudinal analyses of rEZR in the context of MACUSTAR will be possible after the completion of the still ongoing data collection. In future studies, it would also be compelling to compare rEZR profiles to fine-detailed functional testing (e.g. mesopic and scotopic fundus-controlled perimetry) as well as genetic risk profiles. Although the here presented data support the assumption of AMD as a widespread retinal disease, the impact of iAMD features’ topography within a volumetric SD-OCT scan on the rEZR needs to be evaluated in follow-up studies. Given current work on the establishment and implementation of an innovative image analysis tool, it is anticipated to assess a features’ spatial information in a precise and comprehensive manner in the near future. Further limitations are the limited number of participants available in the early AMD group as well as missing follow-up data (V3 visit) for single patients, what might have underpowered results of the test–retest reliability of the rEZR determination. Additionally, as already mentioned above, retinal areas with RPD were not excluded from the analysis what needs to be considered when interpreting the findings of this study. Strengths of this study are the standardized retinal imaging protocol performed with same retinal imaging devices by trained study site personnel, a high image data quality, the highly standardized grading of retinal imaging biomarkers in a central reading center as well as the use of an innovative and in an external data set validated approach for rEZR determination^15^.

In conclusion, this is the first study demonstrating significant rEZR differences across AMD severity stages emphasizing its value as a potential biomarker for photoreceptor degeneration in AMD. We also present benchmark reliability data for assessment of rEZR. Given the systematic exclusion of retinal areas with sub-RPE drusen, this study further supports the assumption of pathophysiological mechanisms beyond typical drusen in AMD. This is worthy of further evaluation considering the need for potential new therapeutic targets and therefore sensitive biomarkers in iAMD. The results of this study warrant a more refined characterization of the rEZR, especially with regards to longitudinal analyses and its prognostic value for disease progression into advanced AMD stages.

## Methods

### The MACUSTAR study

In MACUSTAR—a multicentre, low-interventional natural history study conducted at 20 sites across 7 European countries—subjects with AMD were enrolled from March 2018 to February 2020 (ClinicalTrials.gov Identifier: NCT03349801). The last visit of the last study patient is expected for February 2023. Details on the study design, including inclusion/exclusion criteria have already been published^20,21^. Briefly, based on Ferris et al.^5^, early AMD was defined as presenting with medium-sized drusen (> 63 µm and ≤ 125 µm) in the absence of any AMD pigmentary alterations and any signs of intermediate or late-stage AMD manifestations in both eyes. For iAMD, both eyes had to exhibit large drusen (> 125 µm) and/or any AMD pigmentary abnormalities. In addition, any extrafoveal geographic atrophy (GA) lesion not larger than 1.25 mm^2^ could be present in the fellow eye. Late AMD subjects were required to present bilateral GA of at least 0.1 mm^2^, bilateral neovascular (n) AMD, or nAMD in one eye and GA in the other, while healthy individuals with no signs of early, intermediate or late AMD in both eyes were included as controls^20^.

For all study participants, only one eye was included as a study eye. If both eyes were eligible for the study, the eye with better visual acuity was selected as the study eye. Human research ethics committee approval was obtained at all participating clinical sites prior to study start, complying with all applicable legal regulations as previously described^18^. These committees included University Hospital Bonn ethics committee (384/17), Paris Ouest IV (04/18_2), AIBILI (032/2017/AIBILI/CE), Nova Medical School (13507/2017), London Queen Square Research Ethics Committee (18/LO/0145), Center for Sundhed Glostrup (H-18000126), Comitato Etico Milano (37910/2018), Ospedale San Raffaele (dated 25/10/2018), Radboudumc technology center (2017-3954) and LUMC commissie medische ethiek (L18.055/SH/sh). Participants provided informed consent prior to study recruitment and data collection and this study has been conducted according to the provisions of the Declaration of Helsinki.

Four groups of participants with different disease stages of early, intermediate and late-stage AMD as well as controls were included in the cross-sectional study part consisting of one additional validation visit (V3, day 14 ± 7 days after baseline) scheduled shortly after screening (V1, day − 28 to 0) and baseline (V2, day 0).

### Retinal imaging

Standardized retinal imaging included combined confocal scanning laser ophthalmoscopy (cSLO) [near-infrared reflectance (IR), multicolor, green and/or blue fundus autofluorescence imaging (FAF, Automated Real-Time mode (ART) at least 30 single frames)],spectral-domain optical coherence tomography (SD-OCT, Heidelberg Engineering, Heidelberg, Germany) [20° × 20°, 25 B-scans, distance 240 µm, Automated Real-Time (ART) mode, 4 frames; 30° × 25° enhanced-depth-imaging (EDI) mode, 241 B-scans, distance 30 µm, ART mode, 9 frames] and colour fundus photography (CFP). OCT-angiography (OCT-A) imaging combined both 3 × 3 mm and 6 × 6 mm cube scans, minimum signal strength 8 (Zeiss Cirrus HD-OCT 5000 Angioplex, Zeiss PLEX Elite 9000 swept-source (SS)-OCT) or 20° × 20° and 10° × 10° raster scans (512 B-Scans with 512 A-scans, centered on the fovea, ART 7 mode, Heidelberg Engineering Spectralis OCT-2). Imaging data were transmitted to the GRADE Reading Center (University of Bonn, Germany).

### Image grading

The dense SD-OCT raster scan was primarily assessed for the determination of structural iAMD image features as follows, while other modalities provided complementary and confirmatory information^31^. Reticular pseudodrusen (RPD) were defined as hyperreflective irregularities and elevations above the RPE/Bruch's membrane band in SD-OCT imaging, presenting with medium-reflective mounds or cones at the level of the EZ or between the EZ and the RPE surface. Additionally, RPD were graded to be present when a network of at least five individual lesions with an oval or roundish configuration was detectable in en-face FAF and/or IR imaging^32,33^. Pigmentary abnormalities (PA) were determined as any definite hyper- or hypopigmentary abnormalities associated with sub-RPE drusen, not being related to other known disease entities, on CFP imaging^5^. Pigment epithelial detachment (PED) was defined as a RPE elevation with a basal diameter of at ≥ 1000 µm and a height of ≥ 200 µm, as measured from the inner edge of Bruch's membrane to the outer edge of the RPE band in SD-OCT imaging^34^. Refractile deposits were determined either by a laminar intense hyperreflectivity (≥ 100 µm) at Bruch’s membrane level or a pyramidal structure on the outer retina ("ghost drusen") in SD-OCT. At the corresponding location, a glistening and yellow-shining appearance by CFP, a hyperreflective signal alteration in IR and/or a mildly increased or mottled FAF signal determined the presence of any refractile lesion^35,36^. Acquired vitelliform lesions were defined as hyperreflective, amorphous material confined to the subretinal space and on top of a sub-RPE druse in SD-OCT imaging, typically associated with an increased FAF image signal^37,38^. Quiescent (q) CNV was assumed to be present in the case of a "double layer sign" or a "shallow irregular RPE elevation" (SIRE) without any signs for exudative activity in SD-OCT. OCT-angiography showing a neovascular network with a flow signal at sub-RPE level at the corresponding retinal position confirmed qCNV presence^39,40^.

### Deep-learning based retinal layer segmentation

To delineate the retinal pigment epithelial drusen complex (RPEDC), a multilayer segmentation of each SD-OCT B-scan was performed, based on the retinal layer definitions by Staurenghi et al.^13^. Herein, a deep-learning (DL) based approach with a Deeplabv3-ResNet50 model (Deeplabv3 model with a ResNet-50 backbone) was applied encoding multi-scale contextual information and global context providing excellent performance for semantic segmentation tasks^23,41^. The combined training and validation set (randomly split in a patient-wise manner) comprised of 90 single B-scans from 10 healthy eyes, 540 single B-scans from 80 eyes with intermediate AMD and 342 B-scans from 38 eyes with advanced AMD (30° × 25° field size) in which retinal layer segmentation was performed manually. Herein, various OCT B-scans at the same retinal location relative to the fovea were assessed for manual segmentation across all eyes.

### Determination of the rEZR

**Figure 4:**
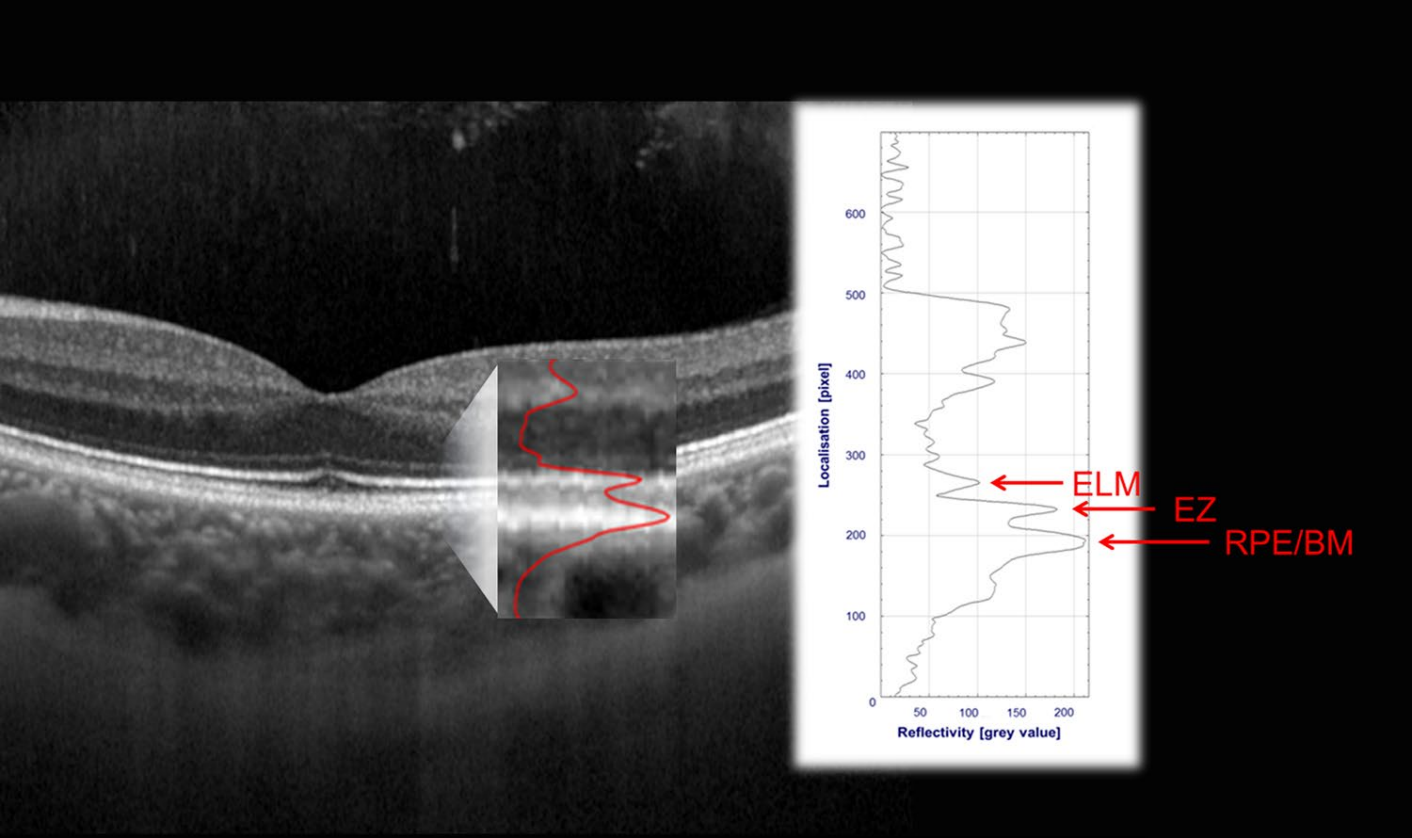
Quantification of SD-OCT based outer retina reflectivity in corresponding reflectivity profiles in a representative healthy case. Each hyperreflective outer retinal band (e.g., the external limiting membrane (ELM), the ellipsoid zone (EZ) or retinal pigment epithelial/Bruch’s membrane complex (RPE/BM)) specifically corresponds to a peak in the reflectivity profile (see red overlay on SD-OCT image detail). Peak detection and assessment of the peak height in the reflectivity profile allows for reflectivity quantification.

Reflectivity profiles were used to quantify the rEZR as the ratio of the EZ peak reflectivity to the peak reflectivity of the external limiting membrane (ELM), see Fig. 4. Here, the rEZR was determined in a semi-automated process in SD-OCT raw images, which allowed for quantitative assessment of "native" image reflectivities, using MatLab (The MathWorks, Version 9.5 Natrick, MA, USA), as following^10,15,42,43^. Segmentation coordinates (as determined by DL, see above) were superimposed to the OCT raw images (i.e., native, non-logarithmic transformed data) and used for straightening of each OCT B-scan along with the coordinates of the RPE which finally enables accurate rEZR determination even in eyes with pronounced curvatures of the posterior pole. Within every single B-scan of the OCT raster scan, the rEZR data was obtained at the adjoining region of interests (ROI) in corresponding reflectivity profiles by assessing the ratio of the EZ to the ELM peak intensity (dynamic range of grey values: 0–1 [arbitrary units, AU]). The width of each ROI was set at 10 pixels (~ 120 µm in high-speed Spectralis OCT imaging) along the image x-axis. The peak intensities were automatically determined in the reflectivity profiles, specifically for each retinal ROI location, so-called EZ and ELM subregions^15^. Sub-regions were pre-defined on OCT raster scans following the same image protocol obtained from healthy individuals. To avoid any optimization bias, these sub-regions were defined using the fellow eyes of the control group (n = 56), as part of the cross-sectional MACUSTAR study. These OCT data were excluded from all other analyses. In assessing the rEZR, the EZ reflectivity was related to the ELM reflectivity based on previous reports^14,44^. These postulated the ELM reflectivity to be stable across a wide eccentricity and to be present in the foveal region which enables rEZR determination across entire SD-OCT B-scans. Further, the ELM is as a non-neural layer and assumed to be one of the retinal layers undergoing least reflectivity alterations with increasing age or (early) stages of degeneration.

For the purpose of this study, retinal areas with sub-RPE drusen were automatically excluded to account for the impact of structural changes within the outer retina on the localized reflectivity profiles^14,44,45^. Herein, each ROI with at least one pixel along the image x-axis, at which the distance between the RPE and Bruch's membrane was identified to be at least 15 pixels (~ 100 µm on the image y-axis in high-speed Spectralis OCT imaging), was assumed to cover a sub-RPE drusen and excluded from the following analysis of this study. By analogy, lesions of macular neovascularizations (MNV) in nAMD eyes were therefore also automatically excluded from further analysis. Given the nature of GA, there is a loss of EZ and ELM in GA areas impeding rEZR determination, which were therefore excluded from further analysis.

### Data analysis

All presented analyses were exploratory, and p-values were not adjusted for multiple testing. Analyses were performed using the statistical software R, version 4.0.3.

To investigate associations between rEZR and AMD severity stage, a linear mixed-effects model was used with patient as a random effect term. Outcome variables were mean rEZR [arbitrary unit, AU] determined in each dense SD-OCT volume raster scan of each study participant excluding drusen areas, and areas with GA or MNV lesions. After exclusion of these retinal areas, a mean of 7020 data points for each study patient and each visit were included in the analyses and were adjusted for age, sex and eccentricity, with eccentricity entering the model as a spline (B-spline of degree 2) of distance to the center. High risk features (e.g. presence of RPD, PED, GA in fellow eyes, PA, vitelliform material, refractile deposits, qCNV and RPEDC volume) within the iAMD group were considered both separately, and in a model, including all biomarkers. p-values < 0.05 were considered significant.

Intra-class Correlation Coefficients (ICC (1,1)) were computed to assess repeatability between V1 and V3 measurements^46^. Repeatability was assessed both for the overall cohort and separately for each AMD severity group. Deming regression was used to evaluate potential systemic differences between the screening (V1) and validation (V3) visit. Bland–Altman plots with 95% limits of agreement visualized the differences between rEZR values at V1 and V3. In these plots, observations above or below the mean difference ± 1.96 times the standard deviation of the differences were further examined for possible reasons of low agreement.

## Supplementary Information

Supplementary Table S1.

## Data Availability

The datasets generated during and/or analyzed during the current study are available from the corresponding author on reasonable request.
