# Supplementary material for: Relative ellipsoid zone reflectivity and its association with disease severity in age-related macular degeneration: a MACUSTAR study report
Source: Sci Rep. 2022 Sep 2;12:14933. doi: 10.1038/s41598-022-18875-5 (PMC9440143; doi:10.1038/s41598-022-18875-5)
Supplement: Supplementary file 1 — Supplementary Table S1. [file 41598_2022_18875_MOESM1_ESM.docx]

**SUPPLEMENTAL TABLE S1:** Detailed results of the separate linear-mixed model analyses of the impact of iAMD high-risk features on the rEZR in eyes with iAMD as presented with coefficient estimates, standard error and p-value, respectively.

|  | **Coefficient estimates** | **Standard error** | **p-value** |
| --- | --- | --- | --- |
| (Intercept) | 80.8 | 11.14 |  |
| Age at baseline [years] | -0.72 | 0.16 | <0.0001 |
| Gender [male] | 3.28 | 2.43 | 0.181 |
| bs(Eccentricity) [°] | -18.93 | 10.8 | <0.0001 |
| **Presence of RPD** | -8.84 | 2.92 | 0.0028 |
|  |  |  |  |
| (Intercept) | 92.5 | 11.14 |  |
| Age at baseline [years] | -0.91 | 0.16 | <0.0001 |
| Gender [male] | 4.21 | 2.48 | 0.0912 |
| bs(Eccentricity) [°] | -18.93 | 0.11 | <0.0001 |
| **Presence of PED** | -9.63 | 5.57 | 0.0855 |
|  |  |  |  |
| (Intercept) | 89.29 | 1.12 |  |
| Age at baseline [years] | -0.86 | 0.16 | <0.0001 |
| Gender [male] | 3.49 | 2.48 | 0.1609 |
| bs(Eccentricity) [°] | -18.93 | 0.11 | <0.0001 |
| **Presence of GA in FE** | -10.49 | 6.34 | 0.0998 |
|  |  |  |  |
| (Intercept) | 92.24 | 11.13 |  |
| Age at baseline [years] | -0.86 | 0.16 | <0.0001 |
| Gender [male] | 4.06 | 2.44 | 0.0981 |
| bs(Eccentricity) [°] | -18.93 | 0.11 | <0.0001 |
| **Presence of PA** | -6.08 | 2.34 | 0.0104 |
|  |  |  |  |
| (Intercept) | 88.18 | 11.28 |  |
| Age at baseline [years] | -0.85 | 0.16 | <0.0001 |
| Gender [male] | 4.07 | 2.48 | 0.103 |
| bs(Eccentricity) [°] | -18.93 | 0.11 | <0.0001 |
| **Presence of vitelliform material** | -7.89 | 5.93 | 0.186 |
|  |  |  |  |
| (Intercept) | 89.02 | 11.23 |  |
| Age at baseline [years] | -0.86 | 0.16 | <0.0001 |
| Gender [male] | 3.58 | 2.48 | 0.15 |
| bs(Eccentricity) [°] | -18.93 | 0.11 | <0.0001 |
| **Presence of refractile deposits** | -10.51 | 6.93 | 0.131 |
|  |  |  |  |
| (Intercept) | 89.43 | 11.2 |  |
| Age at baseline [years] | -0.86 | 0.16 | <0.0001 |
| Gender [male | 3.32 | 2.49 | 0.1835 |
| bs(Eccentricity) [°] | -18.93 | 0.11 | <0.0001 |
| **Presence of qCNV** | -13.27 | 7.74 | 0.0885 |
|  |  |  |  |
| (Intercept) | 92.95 | 11.47 |  |
| Age at baseline [years] | -0.91 | 0.16 | <0.0001 |
| Gender [male] | 3.37 | 2.47 | 0.175 |
| bs(Eccentricity) [°] | -18.94 | 0.11 | <0.0001 |
| **RPEDC volume [mm³]** | -24.52 | 17.35 | 0.159 |

Abbreviations: Intermediate age-related macular degeneration (iAMD), relative ellipsoid zone reflectivity (rEZR), reticular pseudodrusen (RPD), pigment-epithelium detachment (PED), geographic atrophy (GA), fellow eye (FE), pigmentary abnormalities (PA), quiescent choroidal neovascularization (qCNV), retinal-pigment-epithelium complex (RPEDC) volume. Eccentricity is included as a b-spline with degree 2.
